# Identification of Nucleolin as New ErbB Receptors- Interacting Protein

**DOI:** 10.1371/journal.pone.0002310

**Published:** 2008-06-04

**Authors:** Ayelet Di Segni, Keren Farin, Ronit Pinkas-Kramarski

**Affiliations:** Department of Neurobiology, Tel-Aviv University, Ramat-Aviv, Israel; University of Oldenburg, Germany

## Abstract

**Background:**

The ErbB receptor tyrosine kinases are major contributors to malignant transformation. These receptors are frequently overexpressed in a variety of human carcinomas. The role of the ErbB receptors and their ligands in carcinomas and the mechanism by which their overexpression leads to cancer development is still unclear. Ligand binding to specific ErbB receptor is followed by receptor dimerization, phosphorylation and recruitment of SH2 containing cytoplasmic proteins, which initiate the cascade of signaling events. Nevertheless, increasing data suggest that there are non-phosphorylated receptor–substrate interactions that may affect ErbB-mediated responses.

**Methodology/Principal Findings:**

In the present study, using GST-ErbB4 fusion protein pull down assay and mass spectroscopic analysis, we have found the ErbB receptors interact with nucleolin via their cytoplasmic tail. Nucleolin is a ubiquitous, nonhistone, nucleolar, multifunctional phosphoprotein that is also overexpressed in cancer cells. Our results demonstrate that overexpression of ErbB1 and nucleolin may lead to receptor dimerization, phosphorylation and to anchorage independent growth.

**Conclusions/Significance:**

The oncogenic potential of ErbB depends on receptor levels and activation. Our results suggest that nucleolin may affect ErbB dimerization and activation leading to enhanced cell growth.

## Introduction

The ErbB subfamily of receptor tyrosine kinase contains four members: the epidermal growth factor (EGF) receptor (also called ErbB-1) [1], Neu/HER-2/ErbB-2 [2]–[4], HER-3/ErbB-3 [5], [6] and HER-4/ErbB-4 [7]. These tyrosine kinase receptors possess a large glycosylated extracellular domain to which the ligand binds, a single hydrophobic transmembrane region and a cytoplasmic domain, which carry the tyrosine kinase activity and regulatory phosphorylation sites [8]. The ErbB receptors are expressed in various tissues of epithelial, mesenchymal and neuronal origin. Under normal physiological conditions, activation of the ErbB receptors is controlled by the spatial and temporal expression of their ligands, which are members of the EGF family of growth factors [9]. Ligand binding to ErbB receptors induces the formation of receptor homo- and heterodimers and activation of the intrinsic kinase domain, resulting in phosphorylation on specific tyrosine residues within the cytoplasmic tail. These phosphorylated residues serve as docking sites for a range of proteins, the recruitment of which leads to the activation of intracellular signaling pathways [10].

Protein-protein interactions between domains and specific peptide motifs are key principle of signaling events. Sequence containing phosphotyrosine interacts with cognate SH2 or PTB domains [11]. In addition, increasing data suggest that there are non-phosphorylated receptor–substrate interactions. For instance, eps8, a 92 kDa SH3 domain containing protein, has been identified as a substrate for the EGFR based on its association with the EGFR juxtamembrane domain after EGF stimulation [12]. Furthermore, the association of a nuclear localizing zinc-finger protein, ZPR1, with the cytoplasmic tyrosine kinase domain of the EGFR, is decreased by EGF stimulation [13]. Another example for receptor–substrate interaction was demonstrated for Ebp1 association with the juxtamembrane domain of ErbB-3 in a tyrosine kinase-independent manner. Ebp1 dissociates from ErbB-3 after NRG activation and translocates to the nucleus [14]. It was also demonstrated that Erbin PDZ domain binds to the c-terminal end of ErbB2 receptor and regulates its activity [15]. Moreover, it was also demonstrated that ErbB4 binds PDZ domain-containing proteins that affect ErbB4 localization and signaling [16]. Using a glutathione-S-transferase (GST) fusion protein with ErbB4 cytoplasmic tail and pull down assay we identified nucleolin as a new protein that interacts with ErbB proteins.

Nucleolin is an abundantly expressed acidic phosphoprotein of exponentially growing cells. It is involved in the control of transcription of ribosomal RNA (rRNA) genes by RNA polymerase I, in ribosome maturation and assembly, and in nucleocytoplasmic transportation of ribosomal components [17]. Nucleolin is a ubiquitous, nonhistone nucleolar phosphoprotein, present in abundance at the dense fibrillar and granular regions of nucleolus [18], [19]. Intact nucleolin is the major species and represents 5% of nucleolar protein in dividing cells. In nondividing cells, degraded forms of various molecular size are predominantly expressed due to autodegradation [20]. The protein would therefore appear to be involved in fundamental aspects of transcriptional regulation, cell proliferation, and growth. Recently it was demonstrated that nucleolin, is a multifunctional shuttling protein present in nucleus, cytoplasm, and on the surface of some types of cells [17]. In the present study we demonstrate that ErbB proteins and nucleolin interact. This interaction leads to receptor dimerization and activation as well as to colonies growth on soft agar. We therefore suggest that the cross talk between nucleolin and ErbB proteins may be related to tumor growth.

## Results

### ErbB receptors interact with nucleolin

**Figure 1:**
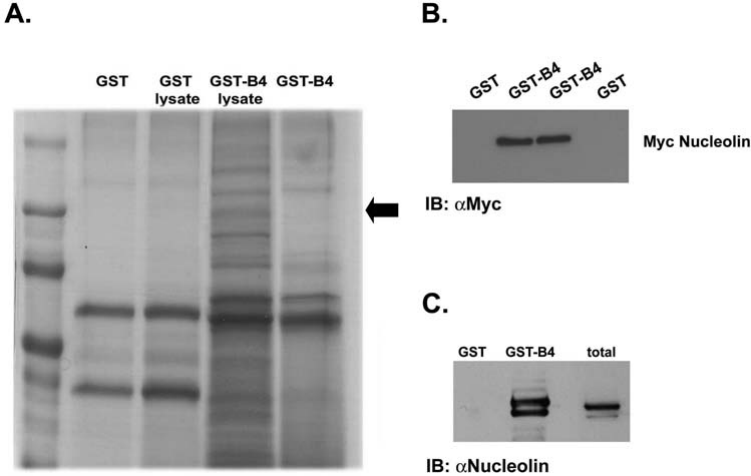
Nucleolin binds the cytoplasmic tail of ErbB4. (A) SDS-PAGE of Coomassie blue–stained proteins isolated from PC12 cell extracts, loaded on GST-ErbB4 agarose affinity matrix or control GST agarose matrix. The arrow indicates a specific 110-kD band, which was identified as nucleolin by mass spectroscopy. (B) COS7 cells were transiently transfected with expression vector of Myc-Nucleolin. Cell lysates were incubated with immobilized GST-ErbB4 or GST. Proteins retained on the beads were resolved by SDS-PAGE and then processed for Western blot using anti-Myc antibodies. (C) Cell lysates prepared from Du145 cells were incubated with immobilized GST-ErbB4 or GST. Eluates from GST-ErbB4 and GST control affinity matrices were resolved by SDS-PAGE and then processed for Western blot using a monoclonal mouse anti-nucleolin antibody.

**Figure 2:**
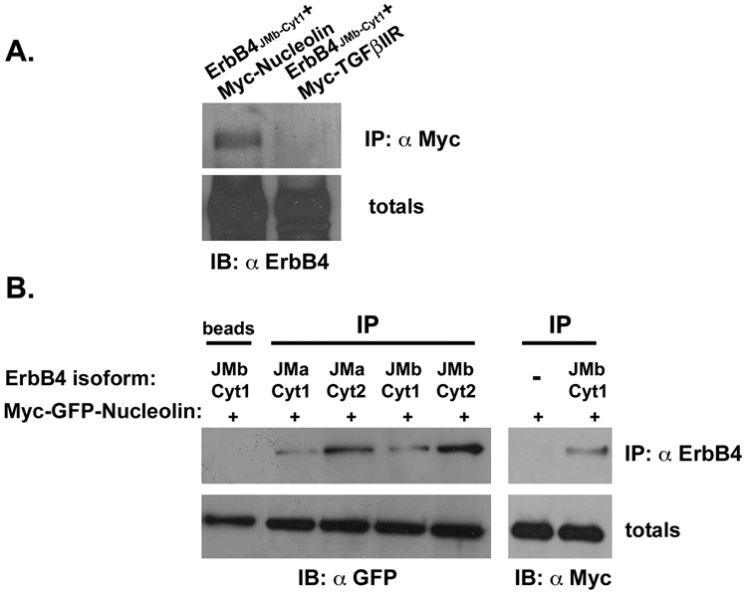
Nucleolin binds to the four ErbB4 isoforms. (A) COS7 cells were transiently co-transfected with expression vector of ErbB4 and either Myc-Nucleolin, or Myc-TGFβIIR. Cell lysates were subjected to immunoprecipitation with anti-Myc antibodies and immunoblotted with anti-ErbB4 antibodies, as a control the total cell lysates were reacted with anti-ErbB4 antibodies. (B) COS7 cells were transiently co-transfected with or without expression vector of each of the ErbB4 receptor isoforms and Myc-GFP-Nucleolin. Cell lysates were subjected to immunoprecipitation with anti-ErbB4 antibodies. The immunoprecipitated proteins as well as total cell lysates were immunoblotted with anti-GFP antibodies or with anti Myc antibodies as indicated.

To identify new ErbB4 interacting proteins we used a pull down assay. A chimeric fusion protein of GST and ErbB4 cytoplasmic tail (GST-ErbB4) was constructed and used as a bait to pull down ErbB4 binding proteins. Affinity chromatography of PC12 cell extracts on a GST-ErbB4 affinity matrix revealed a major ErbB4-binding band at a molecular mass of 110 kD (Figure 1A). Mass spectrometry analysis indicated that the 110-kD band is nucleolin. The identification of the 110-kD protein as nucleolin was confirmed by immunoblotting. First, cell extracts were prepared from COS7 cells transfected with the expression vector encoding Myc-Nucleolin. Lysates were mixed with the GST-ErbB4 beads or with GST beads as a control. A monoclonal anti-Myc antibody, revealed a major 110-kD band in the GST-ErbB4-bound material (Figure 1B). This band was not present in eluates from the control GST matrix. Second, the interaction was assayed on endogenous nucleolin in cell extracts prepared from DU145 prostate cancer cells. As shown in Figure 1C, GST-ErbB4 but not GST pulled down endogenous nucleolin. To further substantiate the results we also performed co-immunoprecipitation experiments. Myc-tagged nucleolin was coexpressed with ErbB4 expression vector. Cell extracts were precipitated with anti-Myc monoclonal antibodies and blotted with anti-ErbB4 polyclonal antibodies (Figure 2A). As shown, nucleolin co-immunoprecipitated ErbB4 protein. As a control we used Myc-TGFβIIR, which did not precipitated ErbB4. Taken together, these results show that the cytoplasmic tail of ErbB4 can specifically interact with nucleolin. Four structurally and functionally distinct ErbB4 isoforms been identified. One pair of isoforms differs within their extracellular juxtamembrane domains (JMa and JMb). These juxtamembrane ErbB4 isoforms are either susceptible or resistant to proteolytic processing that release a soluble receptor ectodomain. Another pair of ErbB4 isoforms differs within their cytoplasmic tails (JM-cyt1 or JM-cyt2) [21]. Since four ErbB4 isoforms exist, we next examined whether nucleolin can interact with the different ErbB4 isoforms. COS7 cells were co-transfected with expression vectors of Myc-GFP-Nucleolin and either ErbB4-JMa-cyt1, ErbB4-JMa-cyt2, ErbB4-JMb-cyt1 or ErbB4-JMb-cyt2. As shown in Figure 2B, all ErbB4 isoforms precipitated with nucleolin.

**Figure 3:**
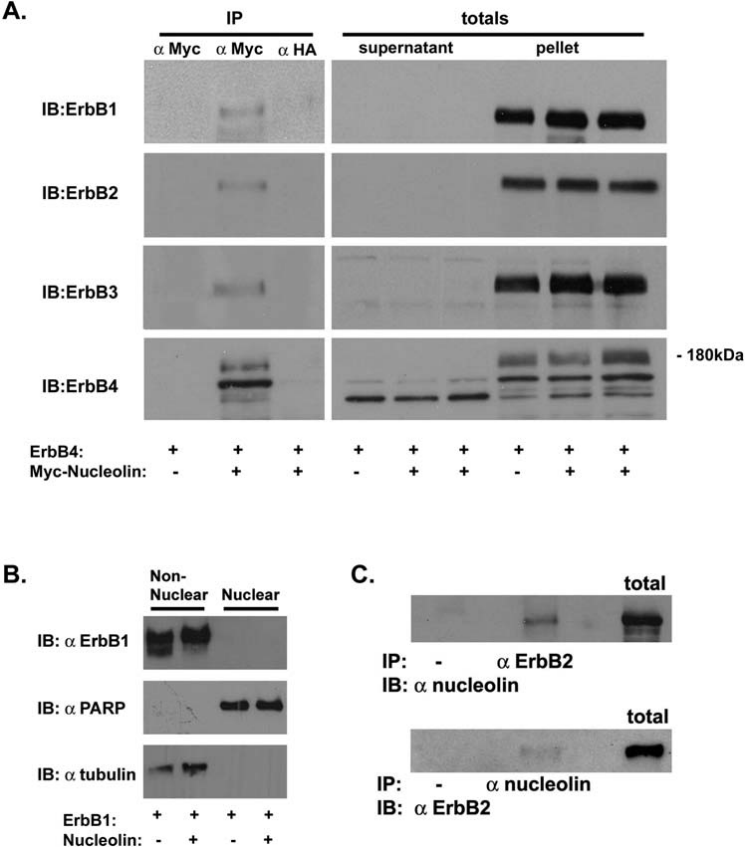
Nucleolin binds to the four ErbB receptors. (A) COS7 cells were transiently co-transfected with expression vector of each of the ErbB receptors and Myc-Nucleolin. Cell lysates were separated into cytosolic (supernatant) and total membrane (pellet) fractions. The pellet fraction was subjected to immunoprecipitation with anti-Myc (nucleolin) or anti-HA (control) antibodies and immunoblotted with specific ErbBs antibodies. Total pellet and supernatant extracts are shown in the right lanes of each panel. (B) COS7 cells were transiently co-transfected with expression vector of ErbB1 and Myc-Nucleolin. Nuclear and non-nuclear cell extracts were immunoblotted with anti-ErbB1 antibodies. As control for the fractionation purity Blots were reacted with anti-PARP antibodies (as a nuclear marker) and anti-tubulin antibodies (as a cytosolic marker). (C) Cell lysates prepared from SKBR3 cells were subjected to immunoprecipitation with either anti-nucleolin or anti ErbB2 antibodies and immunoblotted with either anti- ErbB2 or anti-nucleolin antibodies as indicated, as a control total cell lysates are shown in the right lane of each panel.

To determine whether nucleolin interacts with other ErbB receptors family members we expressed each of the ErbB proteins with or without Myc-tagged Nucleolin in COS7 cells. Lysates of transfected cells were fractionated into supernatant and pellet and immunoprecipitated with an anti-Myc antibody and immunoblotted with individual specific antibodies against ErbB1, ErbB2, ErbB3, and ErbB4. Figure 3A demonstrates that the four ErbB receptors were detected in immunoprecipitates from the pellet (insoluble fraction) of cells co-expressing nucleolin and the receptors, suggesting that all ErbB receptors associate with nucleolin. Moreover, nuclei fractionation of transfected cells reveals that ErbB1, either in the presence or in the absence of nucleolin, is not detected in the nucleus (Figure 3B), indicating that ErbB1/nucleolin interaction occurs mainly in the cell membrane. In addition, using SKBR3 breast cancer cells that express high levels of ErbB2 receptors we demonstrated that endogenous nucleolin precipitated endogenous ErbB2 and vice versa (Figure 3C), indicating, that the interaction may occur also in cells endogenously expressing these proteins.

### Nucleolin induces ErbB receptor phosphorylation

**Figure 4:**
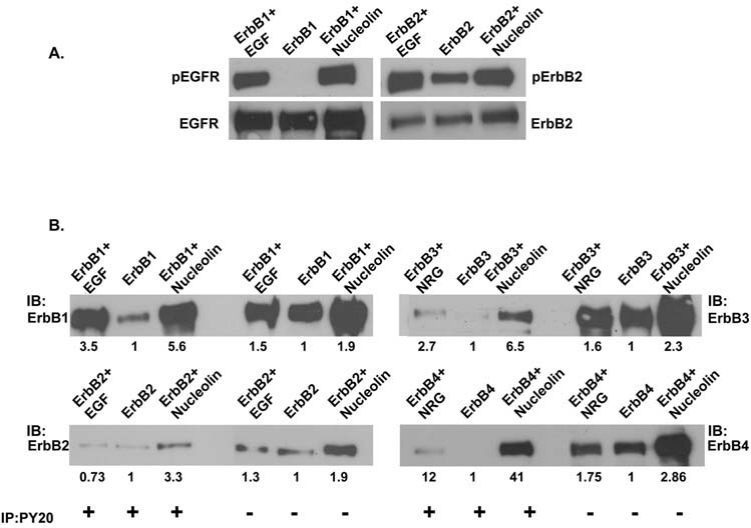
Nucleolin induces ErbB phosphorylation in a ligand independent manner. (A) COS7 cells were transiently co-transfected with expression vector of either ErbB1 or ErbB2 receptors alone or with Myc-Nucleolin. Following 30 min serum deprivation, cells were either untreated or treated with EGF 100 ng/ml for 5 min. Cell lysates were immunoblotted with anti-phosphorylated EGFR or anti-phosphorylated ErbB2 antibodies respectively. As control, lysates were immunoblotted with anti-EGFR or anti-ErbB2 antibodies. Note that at time 0 phosphorylated receptors are detected in cells expressing nucleolin and EGFR or ErbB2. (B) COS7 cells were transiently co-transfected with expression vector of each of the ErbB receptors and Myc-Nucleolin. Following 30 min serum deprivation cells were untreated or treated with either EGF 100 ng/ml or NRG 100 ng/ml for 5 min as indicated. Cell lysates were subjected to immunoprecipitation with anti-phosphotyrosine antibodies (PY20) and immunoblotted with specific ErbB antibodies. Note that in untreated cells, phosphorylated receptor is detected in cells expressing nucleolin and ErbB receptor. The values represent fold induction compared to the receptor levels in untreated cells (1).

To determine whether nucleolin affects ErbB tyrosine phosphorylation we coexpressed ErbB proteins and nucleolin in COS-7 cells. Surprisingly, when nucleolin was coexpressed with ErbB1 it induced receptor phosphorylation even in the absence of the ligand, EGF. This was evident using anti-phosphorylated EGFR antibodies that recognize phosphorylated tyrosine 1068 of the EGFR (Figure 4A). Similarly, coexpression of nucleolin and ErbB2 resulted in ErbB2 phosphorylation as detected using anti-phosphorylated ErbB2 antibodies that recognize phosphorylated tyrosine 1248 on ErbB2 (Figure 4A). To further evaluate the effect of nucleolin on ErbB phosphorylation, lysates of transfected cells were immunoprecipitated with anti-phosphotyrosine antibody and immunoblotted with individual specific antibodies against ErbB receptors. As control for receptor phosphorylation, cells were stimulated with EGF (100 ng/ml) or NRG (100 ng/ml) as indicated. The results demonstrate that nucleolin induces receptor phosphorylation of all ErbB receptors even the ErbB3 that has no kinase activity (Figure 4B). These results suggest that nucleolin ErbB interaction may have functional role, as it affects receptor phosphorylation.

### Nucleolin enhances ErbB1 receptor dimerization

**Figure 5:**
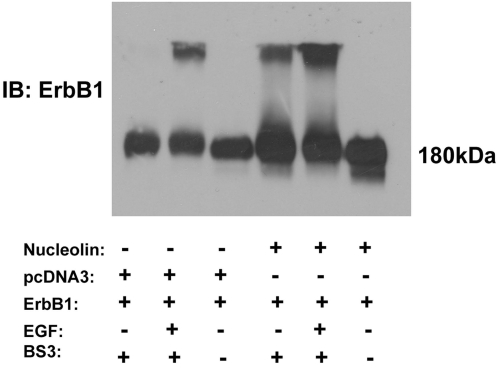
Nucleolin induces ErbB1 dimerization. COS7 cells were transiently co-transfected with expression vector of ErbB1 receptors alone or with Myc-Nucleolin. Following 30 min serum deprivation cells were untreated or treated with EGF 100 ng/ml for 5 min. Cell lysates were incubated with BS^3^ crosslinker (2 mM) for 1 h and immunoblotted with anti-EGFR antibodies. Note that in the presence of nucleolin receptor dimmers appears to be similar to the level of receptor dimmers induced by EGF in the absence of nucleolin.

To address the possibility that nucleolin induces the competence of ErbB receptor dimerization and trans-auto-phosphorylation, we examined the effect of nucleolin on receptor dimerization. Using covalent crosslinking experiment we demonstrated that nucleolin induced ErbB1 dimerization in a ligand independent manner (Figure 5). A similar phenomenon was observed when addressing EGF-induced homodimerization of ErbB-1 as a control. These observations may suggest that the receptor phosphorylation induced by nucleolin may result due to nucleolin-mediated receptor dimerization.

### Nucleolin overexpression reduces receptor disappearance and induces anchorage-independent growth in ErbB1 overexpressing Rat-1 cells

**Figure 6:**
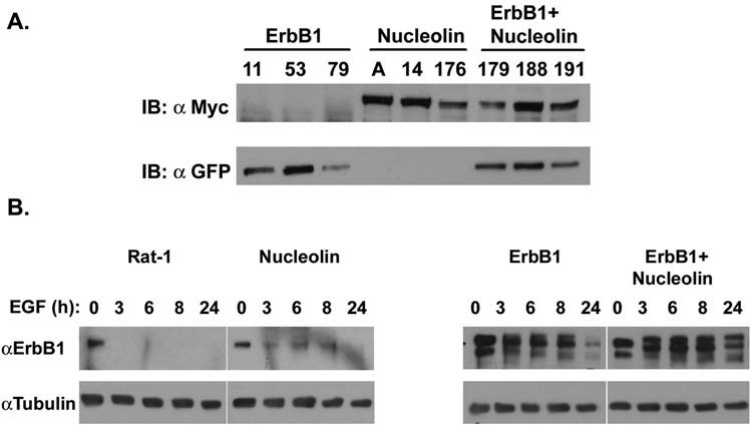
Nucleolin reduces ErbB1 disappearance. (A) Total cell lysates of Rat-1 cells stably expressing ErbB1, nucleolin or ErbB1 and nucleolin were immonoblotted with anti-Myc to recognize Myc-Nucleolin or anti-GFP to identify GFP-ErbB1. (B) Rat-1 cells stably expressing empty vector, ErbB1, nucleolin or ErbB1 and nucleolin were grown in 6 wells plates (10^6^ cells/well). Cells were deprived of serum for 24 h then treated with EGF 100 ng/ml for the indicated time periods. Total cell lysates were analyzed by Western blot, using anti-ErbB1 antibodies. As control total cell lysate was immunoblotted with anti-tubulin antibodies. Note that in the presence of nucleolin the EGFR is more stabilized following EGF stimulation.

**Figure 7:**
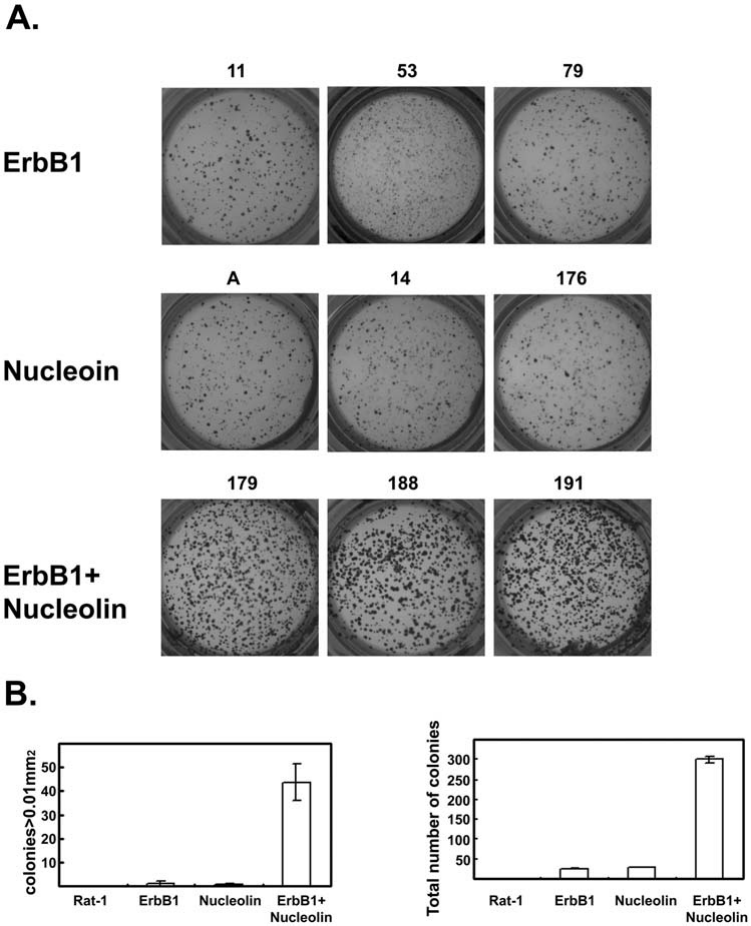
Nucleolin and ErbB1- induced anchorage-independent growth. (A) Rat-1 cells stably expressing empty vector (not shown), ErbB1, nucleolin or ErbB1 and nucleolin were seeded in soft agar (6000 cells/well in 96 well plates) in medium containing 10% FBS, 0.3% agar, in the presence of 100 ng/ml EGF. The extent colony formation was determined 2 weeks later. Cells were dyed with MTT and the wells were photographed and colonies were counted. (B) Results were quantified using image analyzer program Image pro-Plus. The results are presented as total number of colonies and size of colonies. Note that nucleolin+ErbB1 but not each protein alone induced more and larger colonies. Results are the mean±SD 6 determinations. Each experiment was repeated at least three times with 3 different clones, with similar results.

In order to further examine the effect of nucleolin on ErbB1-mediated responses we prepared Rat-1 cells stably transfected to express ErbB1, nucleolin or ErbB1 and nucleolin. The expression levels of ErbB1 or nucleolin in the selected clones is shown in Figure 6A. Next, we examined the effect of EGF on receptor levels in the various cell lines. The Rat-1 cell lines were stimulated with EGF (100 ng/ml) for the indicated time and cell extracts were subjected to Immunoblot analysis. As shown in Figure 6B, EGF induced a time dependent reduction in ErbB1 levels which was significantly lower in cells overexpressing nucleolin. These results indicate that nucleolin may affect receptor levels either by reducing receptor degradation or by increasing receptor formation. In order to assess effects of ErbB1 and nucleolin on colony formation induced by EGF, Rat-1 stable clones were plated in soft agar and maintained in culture for 14 days, in the presence of 100 ng/ml EGF. The number and size of colonies were then estimated from three individual clones. Results of a typical experiment are shown in Figure 7. Rat-1 cells overexpressing either ErbB1 or nucleolin formed relatively small colonies. However, Rat-1 cells overexpressing ErbB1 and nucleolin formed relatively large colonies in soft agar. Moreover, the number of the colonies was significantly higher in ErbB1 and nucleolin overexpressing cells compared to the ErbB1 or nucleolin overexpressing cells (p<0.001). Pools of mock-transfected Rat-1 cells were plated in soft agar as a control. The mock-transfected Rat-1 cells, even in the presence of EGF, were unable to proliferate in the absence of adhesion, and formed no colonies in soft agar (data not shown). These results indicate that EGF enhanced the anchorage-independent growth of the cells overexpressing both ErbB1 and nucleolin.

## Discussion

We report that nucleolin associates with the cytoplasmic region of all ErbB-4 receptor isoforms. Moreover, our findings demonstrate that nucleolin associates also with the other ErbB family members; ErbB1, ErbB2 and ErbB3. Overexpression of nucleolin with ErbB proteins enhances dimerization and phosphorylation, reduces ligand-induced receptor degradation, and enhances ligand-induced anchorage independent growth. These results indicate that nucleolin may associate with the ErbB receptors and affect their activation even in a ligand independent manner. Furthermore, based on the presented data we suggest that nucleolin may modulate ErbB receptors activities.

The ErbB family of receptor tyrosine kinases consists of four different proteins called EGFR/ErbB1/HER1, ErbB2/Neu/HER2, ErbB3/HER3, and ErbB4/HER4 [22]. Under normal physiological conditions, the ErbB receptors play crucial roles in propagating signals regulating cell proliferation, differentiation, motility, and apoptosis [23]. They are activated by ligand binding, which leads to homo- or heterodimerization followed by trans-phosphorylation of specific tyrosine residues. These phosphorylated tyrosines, in turn, provide recognition sites for cytoplasmic proteins, which link ErbB receptors to downstream signaling transduction cascades such as the MAP kinase pathway. As overexpression of ErbB receptors is often found in several human tumors such as breast, lung, head, and neck [24], their precise role in the cell is of particular biological and pharmacological importance.

Nucleolin is a major constituent of nucleoli in exponentially growing cells [19] and functions in the organization of nucleolar chromatin [25], packaging of pre-rRNA [26], rDNA transcription [27], and ribosome assembly by shuttling between the nucleus and the cytoplasm [28]. It was demonstrated that nucleolin, is a multifunctional protein present in nucleus, cytoplasm, and on the surface of some types of cells [17]. Although mainly characterized as a nucleolar protein, nucleolin also functions as a cell surface receptor where it is associated with the actin cytoskeleton and acts as a shuttling protein between cytoplasm and nucleus [29]. Cell surface nucleolin has been reported to bind lipoproteins, laminin, midkine, L-selectin, lactoferrin and other growth factors [30]–[33]. Moreover, nucleolin was identified as a binding partner for receptor protein tyrosine phosphatase-sigma ectodomain in skeletal muscle [34]. Thus, the present findings of this new interaction between nucleolin and the ErbB cell surface receptors, add another layer to the complexity of nucleolin/ ErbB- mediated functions.

Our study demonstrates that nucleolin not only binds all ErbB receptors family members but can also affect ErbB1, ErbB2 ErbB3 or ErbB4 phosphorylation. Among ErbB family members, ErbB1 and ErbB4 are fully functional receptor tyrosine kinases, whereas ErbB2 does not bind any known ligand and ErbB3 has no intrinsic kinase activity, however, ErbB3 still mediate signaling by heterodimerization with other receptors of the family [22]. Our results demonstrating the ability of nucleolin to induce ErbB3 phosphorylation may be a consequence of heterodimerization with endogenous ErbB1 or ErbB2 expressed in COS7 cells. Thus, overexpressed nucleolin may affect the levels of receptor activation.

Ligand-induced receptor phosphorylation depends on receptor dimerization [10]. However, it was also demonstrated that protein interactions may affect receptor activities. For example, Erbin protein binds to the c-terminal end of ErbB2 receptor and regulates its activity [15]. Nucleolin by interacting with ErbB proteins also affect their activities. It was reasonable to assume that nucleolin affects receptor dimerization and thus its activation. Indeed, using crosslinking experiments we demonstrated that nucleolin induces ErbB1 receptor dimerization in a ligand independent manner. This observation may suggest that nucleolin binding to the cytoplasmic tail of the receptor may affect the ability of the receptor to dimerize.

Similarly to ErbB proteins, nucleolin is abundant in proliferating cancerous cells, and high levels of nucleolin expression are related to poor clinical prognosis for certain types of cancer [35], [36]. The ability of nucleolin to affect receptor dimerization and phosphorylation in a ligand independent manner may partially explain the constitutive activation of ErbB proteins in specific tumors [37]–[39]. In our study we demonstrated that EGF stimulation induced reduction in receptor levels in the mock or ErbB1 expressing cells and slower receptor disappearance in nucleolin expressing cells. In addition, anchorage independent growth in cells expressing nucleolin and ErbB1 was significantly higher compared to cells expressing each of these proteins alone. These may result from extended EGFR signaling in nucleolin expressing cells. Currently we do not know whether the effect of nucleolin on receptor levels results from changes in receptor internalization, degradation or synthesis.

In conclusion, our studies provide the first evidence for the connection between nucleolin and ErbB receptors. These results suggest that nucleolin not only interacts with ErbB proteins but may also affect their activation. It may also provide a new insight into the mechanism by which overexpressed ErbB receptors mediate cell transformation in a ligand independent manner. Nucleolin is mainly expressed in the nucleus. ErbB receptors are mainly expressed in the cell membrane. Interestingly, nuclear localization of EGFR was previously demonstrated and a direct role of EGFR as transcription factor was suggested (reviewed in [40]). Other ErbB family members were also found in the nucleus; ErbB2/HER2 [41], ErbB4/HER4 [42] and ErbB3/HER3 [43]. Nucleolin may conceivably function as a shuttle protein directing ErbB receptors into the nucleus. Our results indicate that the initial interaction is in the cell membrane, however, further studies are needed to explore the exact localization of these proteins following ligand stimulation and the mechanism by which nucleolin affects ErbB activation and ErbB-mediated responses.

## Materials and Methods

### Materials and Buffers

Human recombinant NRGβ was purchased from R&D System Inc. (Oxon, UK). EGF (human recombinant) was purchased from Boehringer Mannheim. Polyclonal rabbit anti- ErbB1, ErbB2, ErbB3, ErbB4 antibodies, monoclonal mouse anti-phosphotyrosine (PY20), mouse anti-GFP (B-2) and mouse anti-nucleolin (C23) were purchased from Santa Cruz Biotechnology (Santa Cruz, CA, USA). Polyclonal rabbit anti phosphorylated ErbB1 (Tyr1068) and phosphorylated ErbB2 (Tyr1248) were purchased from Cell Signaling technology. Monclonal mouse anti-PARP antibodies were from BIOMOL International, LP. Monoclonal mouse anti c-Myc (9E10) and monoclonal anti HA (12C5α) was donated by Dr. Altchuler Yoram the Hebrew University, Israel. All other reagents were from Sigma. HNTG buffer contained 20 mM HEPES (pH 7.5), 150 mM NaCl, 0.1% Triton X-100 and 10% glycerol. Solubilization buffer contained 50 mM HEPES (pH 7.5), 150 mM NaCl, 1% Triton X-100, 1 mM EGTA, 1 mM EDTA, 1.5 mM MgCl_2_, 10% glycerol, 2 mM sodium vanadate, 1 µM phenylmethylsulfonylfluoride, 10 µg/ml aprotanin and 10 µg/ml leupeptin. Pull Down buffer contained 50 mM TRIS-HCl (pH 7.6), 20 mM MgCl2, 150 mM NaCl, 1 mM DTT, 2 mM sodium vanadate, 1 µM phenylmethylsulfonylfluoride, 10 µg/ml aprotanin, 10 µg/ml leupeptin, 0.5% NP40, 5 µg/ml pepstatine and 1 mM Benzamidine. Binding buffer contained 50 mM TRIS-HCl (pH 7.6), 10 mM MgCl_2_, 100 mM NaCl, 0.5 mM DTT and 0.5 mg/ml BSA.

### Cell lines

COS-7, SKBR3 and Rat-1 cell lines were grown in Dulbecco's modified Eagle's (DMEM) supplemented with 10% fetal bovine serum. PC12-ErbB4 cell line [44] was grown in Dulbecco's modified Eagle's supplemented with 10% fetal bovine serum and 10% horse serum. DU145 cell line was grown in RPMI 1640 supplemented with 10% fetal bovine serum. For transient expression cells were transfected using jetPEI (Poly plus transfection, USA). Cell lysates were prepared 48 h following transfection as described. The Rat-1 fibroblast cells were used for stably expressing the ErbB-1 receptor and nucleolin. Expression vector pEGFP-ErbB-1 containing the coding region of ErbB-1 and expression vector pCDNA3-Myc-Nucleolin containing the coding region of nucleolin [45] were introduced by CaPO_4_ transfection into Rat-1 cells either alone or together. The neomycin (G418) resistant colonies were checked for ErbB-1 or nucleolin expression and several colonies were selected for further analysis.

### DNA constructs

The ErbB4 cytoplasmic tail was amplified using the following primers: 5′ TAGACCCGGGAGAAGGAAGAGCATC and 5′ TCGCCCGGGTTATGACACCACAGTATTCCG. The amplified fragment was digested with XmaI and cloned into pGEX-3X expression vector.

A C′ terminally Myc-tagged mouse nucleolin (pMT21-Myc-Nucleolin) was generously provided by Dr. Bacharach E. (Tel-Aviv University, Israel). Myc -Nucleolin cDNA was amplified using the following primers: 5′ CCGGAATTCGCCACCATGGTGAAGCTCGCG and 5′ CGAACCATGATGGCTCGAATCAC. The amplified fragment was digested with EcoRI and XbaI and cloned into pcDNA3 expression vector. In order to generate GFP-fused Nucleolin-Myc expression vector, the pcDNA3-Nucleolin-Myc vector was digested with EcoRI and ApaI and cloned into pEGFP-C2 expression vector. ErbB1-GFP (pEGFP-N2) was generously provided by Dr. Erlich M (Tel-Aviv University, Israel).

### Lysate Preparation, Cell fractionation, Immunoprecipitation and Immunoblotting

Cells were exposed to the indicated stimuli. After treatment, cells were solubilized in lysis buffer. Lysates were cleared by centrifugation. For direct electrophoretic analysis, boiling gel sample buffer was added to cell lysates. For cell fractionation two methods were used. First, cells were homogenized, and the supernatant (soluble proteins) and the pellet (insoluble proteins) fractions were obtained by centrifugation (100,000×g, 30 min, 4°C) [46]. Samples of the pellet fractions were subjected to immunoprecipitation. Second we separated nuclei from the other cell fractions as described previously [47]. Briefly, cells were washed twice with ice-cold phosphate-buffered saline, harvested by scraping with a rubber policeman, and lysed in a lysis buffer (20 mM HEPES, pH 7.0, 10 mM KCl, 2 mM MgCl2, 0.5% Nonidet P-40, 1 mM Na3VO4, 10 mM NaF, 1 mM phenylmethanesulfonyl fluoride, 2 µg/ml aprotinin). After incubation on ice for 10 min, the cells were homogenized by 20 strokes in a tightly fitting Dounce homogenizer. The homogenate was centrifuged at 1,500×g for 5 min to sediment the nuclei. The supernatant was then centrifuged at a maximum speed of 16,100×g for 20 min, and the resulting supernatant formed the non-nuclear fraction. The nuclear pellet was washed three times with lysis buffer to remove any contamination from cytoplasmic membranes. To extract nuclear proteins, the isolated nuclei were resuspended in NETN buffer (150 mM NaCl, 1 mM EDTA, 20 mM Tris-Cl, pH 8.0, 0.5% Nonidet P-40, 1 mM Na3VO4, 10 mM NaF, 1 mM phenylmethanesulfonyl fluoride, and 2 µg/ml aprotinin), and the mixture was sonicated briefly to aid nuclear lysis. Nuclear lysates were collected after centrifugation at 16,100×g for 20 min at 4°C.

For immunoprecipitation, antibodies were first coupled to anti-mouse IgG agarose (for monoclonal antibodies) or protein A-sepharose (for polyclonal antibodies) for 1 h at RT. Then the proteins in the lysate supernatant were immunoprecipitated with aliquots of the beads-antibody complexes for 2 h at 4°C. The immunoprecipitates were washed three times with HNTG, resolved by SDS-polyacrylamide gel electrophoresis (PAGE) through 7.5% gels and electrophoretically transferred to nitrocellulose membrane. Membranes were blocked for 1 h in TBST buffer (0.02 M Tris-HCl pH 7.5, 0.15 M NaCl, and 0.05% Tween 20) containing 6% milk, blotted with 1 µg/ml primary antibodies for 2 h, followed by 0.5 µg/ml secondary antibody linked to horseradish peroxidase. Immunoreactive bands were detected with the enhanced chemiluminescence reagent (Amersham Corp, Buckinghamshire, UK).

### Cross-linking

Cross-linking experiments were performed by addition of 2 mM bis (sulfosuccinimidyl) suberate (BS^3^), to the lysis buffer for 20 min on ice. The chemical crosslinking reaction was stopped by adding 50 mM Glycine and the samples were resolved by SDS-PAGE [48].

### Soft Agar Assay

Cells were seeded at a density of 6000 cells/well in 96 well plates in DMEM containing 10% FBS. The cells were mixed with 0.05 ml (per each well) of 0.33% noble agar, and the mixture was poured onto a layer of 0.05 ml 1% noble agar in DMEM containing 10% FBS. The upper layer of the agar was covered with 0.1 ml of medium. The agar layers contained either PBS (control) or 100 ng/ml EGF. Assays were performed in at least six repeats. The number and sizes of the colonies were estimated on day 14, using a binocular and a light microscope with the image analyzer program Image pro-Plus.

### GST Pull-Down Assay

To characterize proteins that interact with the cytoplasmic tail of ErbB4, a GST-ErbB4 column was generated by absorbing 20 ml of GST-ErbB4 -producing E.coli lysate (resulting from a 1-liter culture) to 1 ml of glutathione-Sepharose (Sigma). A similar column was prepared from GST-producing E. coli. PC12 cell lysate was prepared and a total of 2 mg protein was loaded on GST or GST-ErbB4 columns (in the presence of 0.5 ml binding buffer) for 2 h at 4°C. After the incubation the beads were washed three times with binding buffer. The GST and GST-ErbB4 bound proteins were eluted in boiling sample buffer and resolved by SDS-PAGE through 7.5% gels and either electrophoretically transferred to nitrocellulose membrane, or protein bands on the gel were visualized by Coomassie Blue staining. In the latter case, bands uniquely bound to GST-ErbB4 were further analyzed by Mass Spectrometry using API QSTAR™ Pulsar Hybrid LC/MS/MS System.
